# Mental and Physical Impact of Body Contouring Procedures on Post–Bariatric Surgery Patients

**Published:** 2012-09-12

**Authors:** Devinder Singh, Hamid R Zahiri, Lindsay E Janes, Jennifer Sabino, Jamil A Matthews, Robert L Bell, J. Grant Thomson

**Affiliations:** ^a^Division of Plastic Surgery, Department of Surgery, University of Maryland Medical Center, Baltimore, Md; ^b^Yale University School of Medicine, Division of Bariatric Surgery, New Haven, Conn; ^c^Yale University School of Medicine, Section of Plastic Surgery, New Haven, Conn

## Abstract

**Objective:** The rapid rate of weight loss following bariatric surgery leads to areas of excessive skin that can cause physical ailments and distortion of body image. Dissatisfaction with the excessive skin can lead patients to seek plastic surgery. This study aims to assess the changes in mental and physical quality of life after body contouring procedures in the post–bariatric surgery population. **Methods:** In this cross-sectional study, the 36-Item Short Form Health Survey was given to 104 patients divided into 4 groups consisting of a control group, obese patients, post–bariatric surgery patients, and post–bariatric and –body contouring surgery patients. Scores from each survey question were individually averaged, scaled, and converted to the corresponding 8 scales that make up the 36-Item Short Form Health Survey. Scale comparisons were accomplished by analysis of variance and *t* test. **Results:** Compared with the obese group, both post–bariatric surgery patients and post–body contouring surgery patients had improved quality of life. When comparing the post–body contouring and post–bariatric surgery patients, the post–body contouring group did not show significant quality of life improvement and actually scored significantly lower in 2 measures, Role Emotional and Social Functioning, indicating a decreased mental component of quality of life. When compared with the control group, the post–body contouring surgery group had statistically significant lower scores in 6 of the 8 scales. **Conclusions:** The functional impairment caused by excessive skin following massive weight loss interferes with quality of life. Patients electing to have body contouring after bariatric surgery show decreased quality of life even after plastic surgery compared to those patients who do not.

Of the numerous weight loss options available to the morbidly obese, bariatric surgery is the only treatment modality proven to provide both durable weight loss and improvement of obesity-related comorbidities.^1^ Despite the success of bariatric surgery, the rapid rate of secondary weight loss often leads to distressing deformities including excess skin and malpositioned adipose tissue. In addition to a compromised body image, this redundant skin can cause functional impairment by harboring intertriginous rashes or by interfering with ambulation, maintaining adequate hygiene, and activities of daily living.^2^^,^^3^ Dissatisfaction with this excessive skin often prompts patients to seek body contouring surgery by a plastic surgeon.^4^ As the frequency of bariatric surgical procedures increases, so does the number patients seeking body contouring procedures after massive weight loss from plastic surgeons.

While numerous studies have established that quality of life (QOL) considerably improves for a given patient following bariatric surgery, few have investigated QOL in patients who elect to have body contouring procedures compared with those who do not. We hypothesized that the population of patients who elect to have plastic surgery will show improvement in both the physical and psychosocial elements of QOL because apparent complaint of excessive skin is remedied by body contouring surgery. This study aims to investigate this hypothesis by using the 36-Item Short Form (SF-36) Health Survey.

## MATERIALS AND METHODS

### Patients

All patients were treated at Yale New Haven Hospital between August 2002 and December 2005. All bariatric surgeries in this study were laparoscopic Roux-en-Y gastric bypass performed by the same bariatric surgeon (R.L.B.). All of the body contouring procedures were performed by the same plastic surgeon (J.G.T.). The survey was given to 4 separate groups of patients: group 1 was the control, a sampling of normal population; group 2 consisted of obese patients prior to bariatric surgery; group 3 was composed of post–bariatric surgery patients with established massive weight loss; and group 4 patients were post–bariatric surgery and had undergone at least one body contouring procedure between August 2002 and December 2005. The survey did not assess whether group 4 patients still desired additional plastic surgery procedures.

### Quality–of-life measurement

This study is a cross-sectional study with prior institutional review board approval. To evaluate patients' QOL, we used the SF-36 Health Survey Version 2.0. The SF-36 assesses the physical and mental components of health by measuring 8 scales of lifestyle described in Table 1: physical functioning (PF), role-physical (RP), bodily pain (BP), general health (GH), vitality (VT), social functioning (SF), role-emotional (RE), and mental health (MH). Quality of life as measured by the SF-36 is broken into 2 components: the physical component and the mental component. The 3 scales that contribute to the mental component in order of decreasing validity are MH, RE, and SF. The 3 scales that contribute to the physical component in order of decreasing validity are PF, RP, and BP. Vitality and GH contribute to both components to a lesser degree.^5^

### Statistical analysis

Scores from each of the 36-survey questions were individually averaged, scaled, and converted to the corresponding 8 scales. Each scale was then compared between each subject group using univariate analysis of variance and Gosset's independent 2-tailed *t* test. A *P* < 0.05 was considered to be statistically significant.

## RESULTS

A total of 104 surveys were completed. The number of participants, gender, average age, and mean body mass index for each group can be seen in Table 2. The scaled composite scores for each of the 8 scales are illustrated in Table 3.

Compared with the control group, QOL scores for the obese group were lower in all 8 categories, reaching significance in all except mental health (*P* = 0.23).

When the post–bariatric surgery group was compared to the obese group, the post–bariatric surgery QOL was considerably improved. With the exception of 2 categories, BP and MH, every other category in the post–bariatric surgery group was significantly higher than the obese group. Interestingly, QOL in the post–bariatric surgery group was very similar to that of the control group, only PF and RP were significantly lower (*P* < .001).

Examination of the post–body contouring group compared to the obese values showed increased QOL in all categories, reaching statistical significance in 4 categories (PF, GH, VT, and SF). Comparison of the post–body contouring group with the post–bariatric surgery group did not reveal increased QOL, and even indicated significantly decreased QOL concerning RE (*P* = .0029) and SF (*P* = .0067).

## DISCUSSION

Only recently have physicians begun to investigate the psychosocial impact of bariatric surgery and subsequent changes in the patient's QOL. The literature to date strongly suggests that surgically obtained weight loss is accompanied by substantial improvements in the following areas: social functioning, rates of employment, active lifestyle, sex life, attitudes toward body weight and shape, and substantial normalization of body image.^6^^-^9 Quantitative data for these improvements are determined by utilizing a multitude of surveys, including the SF-36 Health Survey.^4^^,^^7^^,^^8^ No single best patient-reported outcome measure to assess patient QOL after body contouring surgery is available.^10^ The SF-36 was chosen for our study because it is an easy-to-administer and well-studied instrument in bariatric surgery which has been adopted by the International Bariatric Surgery Registry.^8^ The SF-36 has been shown to be valid and reliable over assorted population groups in evaluating general mental and physical health.^5^ Using the SF-36, our data reaffirmed the established trend that after bariatric surgery there is considerable improvement in all 8 scales of the SF-36.^1^^,^^8^^,^^11^

The literature demonstrating improvement in QOL after bariatric surgery does not address how the induced rapid weight loss often results in excessive skin and malpositioned adipose tissue, consequences that are often perceived by patients as negative outcomes.^12^ These undesirable physical transformations can occur in the medial thighs, mid-abdomen, flanks, breasts, buttocks, upper arms, and elsewhere.^2^^,^^13^ Problems from these excessive skin folds, including intertriginous rashes and ulcerations, interference with ambulation and activities of daily living, and compromise of body image, may negatively impact the physical and mental components of QOL.^2^^,^^3^ Therefore, after removal of skin excess, the QOL scales should theoretically improve after body contouring procedures.

Indeed, several studies have demonstrated improvements in QOL associated with body contouring surgery. Coriddi et al^14^ demonstrated significant improvements in functional outcomes, such as difficulty in personal hygiene, difficulty finding clothes, skin irritation, neck pain, and abdominal pain, after body contouring surgery. Song et al^15^ found that body contouring surgery resulted in improved satisfaction with body image and health-related QOL. A recent study by Van der Beek et al^16^ on the impact of reconstructive procedures after bariatric surgery indicated significant improvements in areas including physical appearance, physical functioning, mental well-being, social acceptance, intimacy, and sexuality. Another study by Pecori et al^7^ found that the overall acceptance of body shape and body image awareness after body contouring surgery were similar to levels seen in patients after satisfactory massive weight loss. All these studies indicate either improvements in measures of QOL after a given patient's receive plastic surgery compared to before.

We found that the scales comprising the mental component were lower in the group of patients who received body contouring procedures, indicating impaired QOL. Two contributors to the mental component, RE and SF, were significantly lower than both the control and the post–bariatric surgery group, showing that those patients who elected to have body contouring procedures suffer from worsening inhibited social interactions. In addition, those patients who had body contouring surgery scaled similarly in the physical components of QOL. Of all 8 scales, PF is the best all around measure of physical health.^5^ Our results indicate comparable improvements in PF in the body contoured group compared to the post–bariatric surgery group. We believe these results demonstrate the persistent dissatisfaction of many patients after receiving body contouring surgery and indicate the importance of appropriate education and management of expectations prior to surgery. We believe patients in the post–bariatric surgery group who elected not to undergo body contouring chose so because their QOL was already high, and thus motivation for surgery was low. Patients who did elect to undergo plastic surgery, however, emerged with a lower QOL than those who did not, possibly due to underlying psychological factors in this group surgery cannot change. Thus, we believe our results indicate the importance of counseling and education prior to plastic surgery to ensure appropriate expectations for the patient in regard to scarring and the realistic scope that can be accomplished by surgery.

There are limitations in this study. As with every other QOL study investigating body contouring surgery, our subject pool was fairly small, making assumptions about the general population difficult. Consequently, our power of 0.3 is low, decreasing our chances of finding statistically significant differences. Another limitation is attributed to the inherent qualities of the SF-36. While the SF-36 is validated for general health as well as specific ailments, the results of the survey does not divulge information regarding characteristic weight loss issues such as identifying particular sources of dissatisfaction. Lastly, we did not determine if the post–body contouring group desired additional procedures, a factor that may have influenced survey scores. Despite these limitations, we feel that our study is a good reflection on the QOL of patients at different stages of the bariatric weight loss process and reflects the importance of managing expectations for patients who desire plastic surgery.

## Figures and Tables

**Table 1:** Eight scales of the SF-36 that assess physical and mental components of health

Scale	Measures
Physical Component	
Physical functioning (PF)	Limitations in physical activities due to health problems
Role-physical (RP)	Limitations in usual daily activities due to health problems
Bodily pain (BP)	Pain experienced due to health problems
General health (GH)	General health perceptions
Vitality (VT)	Self-perceived energy and fatigue
Mental Component	
Social functioning (SF)	Limitations in social activities due to physical or emotional problems
Role-emotional (RE)	Limitations in usual daily activities due to emotional problems
Mental health (MH)	Perceived well-being and the presence of any psychological distress

**Table 2:** Patient demographics

Demographic	Control	Obese	Post–bariatric Surgery	Post–body Contouring Surgery
Participants (n)	27	31	30	16
Men (%)	6 (22%)	6 (19%)	5 (17%)	4 (25%)
Women (%)	21 (78%)	27 (81%)	25 (83%)	12 (75%)
Mean age ± SD (y)	36 ± 11.4	42 ± 11.3	45 ± 10.4	45 ± 9.1
Mean BMI ± SD (kg/m^2^)	23.6 ± 2.6	48.9 ± 7.2	32.2 ± 8.7	31.6 ± 7.4

BMI indicates body mass index; SD, standard deviation.

**Table 3:** Average value of all SF-36 scales for each group with comparative P values

Scale Averages					P Values			
Scale	Control	Obese	Post–Bariatric Surgery	Post–Body Contouring Surgery	Obese vs. Post-bariatric	Post–Body Contouring vs Control	Post–body Contouring vs Obese	Post–Body Contouring vs Post—Bariatric Surgery
PF	0.98	0.62	0.89	0.86	.0000*	.0000*	.0000*	.2066
RP	0.99	0.71	0.93	0.80	.0013*	.0000*	.1046	.3565
BP	0.93	0.62	0.82	0.65	.0605	.0401*	.4595	.1069
GH	0.84	0.53	0.85	0.90	.0000*	.9124	.0000*	.4759
VT	0.77	0.58	0.76	0.69	.0040*	.0381*	.0256*	.0853
MH	0.80	0.74	0.80	0.77	.1517	.4681	.3185	.3610
RE	0.96	0.80	0.94	0.84	.0175*	.0023*	.2663	.0029*
SF	0.87	0.65	0.90	0.76	.0003*	.0216*	.0098*	.0067*

BP indicates bodily pain; GH, general health; PF, physical functioning; MH, mental health; RE, role-emotional; RP, role-physical; SF, social functioning; VT, vitality.

*Indicates statistical significance (used *P* < .05).
